# Selected Applications of Chitosan Composites

**DOI:** 10.3390/ijms222010968

**Published:** 2021-10-11

**Authors:** Kunal Pal, Deepti Bharti, Preetam Sarkar, Arfat Anis, Doman Kim, Renata Chałas, Paweł Maksymiuk, Piotr Stachurski, Maciej Jarzębski

**Affiliations:** 1 Department of Biotechnology and Medical Engineering, National Institute of Technology Rourkela, Rourkela 769008, India; deeptibharti94@gmail.com; 2 Department of Food Process Engineering, National Institute of Technology, Rourkela 769008, India; preetamdt@gmail.com; 3 SABIC Polymer Research Center, Department of Chemical Engineering, King Saud University, Riyadh 11421, Saudi Arabia; aarfat@ksu.edu.sa; 4 Department of International Agricultural Technology & Institute of Green BioScience and Technology, Seoul National University, Pyeongchang 25354, Gangwon-do, Korea; kimdm@snu.ac.kr; 5 Department of Oral Medicine, Medical University of Lublin, 20-093 Lublin, Poland; renata.chalas@umlub.pl (R.C.); pawelmaksymiuk@umlub.pl (P.M.); 6 Chair and Department of Pediatric Dentistry, Medical University of Lublin, 20-093 Lublin, Poland; piotr.stachurski@umlub.pl; 7 Department of Physics and Biophysics, Poznan University of Life Sciences, 60-637 Poznań, Poland

**Keywords:** chitosan, active food packaging material, films, biodegradable, food safety, food security

## Abstract

Chitosan is one of the emerging materials for various applications. The most intensive studies have focused on its use as a biomaterial and for biomedical, cosmetic, and packaging systems. The research on biodegradable food packaging systems over conventional non-biodegradable packaging systems has gained much importance in the last decade. The deacetylation of chitin, a polysaccharide mainly obtained from crustaceans and shrimp shells, yields chitosan. The deacetylation process of chitin leads to the generation of primary amino groups. The functional activity of chitosan is generally owed to this amino group, which imparts inherent antioxidant and antimicrobial activity to the chitosan. Further, since chitosan is a naturally derived polymer, it is biodegradable and safe for human consumption. Food-focused researchers are exploiting the properties of chitosan to develop biodegradable food packaging systems. However, the properties of packaging systems using chitosan can be improved by adding different additives or blending chitosan with other polymers. In this review, we report on the different properties of chitosan that make it suitable for food packaging applications, various methods to develop chitosan-based packaging films, and finally, the applications of chitosan in developing multifunctional food packaging materials. Here we present a short overview of the chitosan-based nanocomposites, beginning with principal properties, selected preparation techniques, and finally, selected current research.

## 1. Introduction

**Figure 1:**
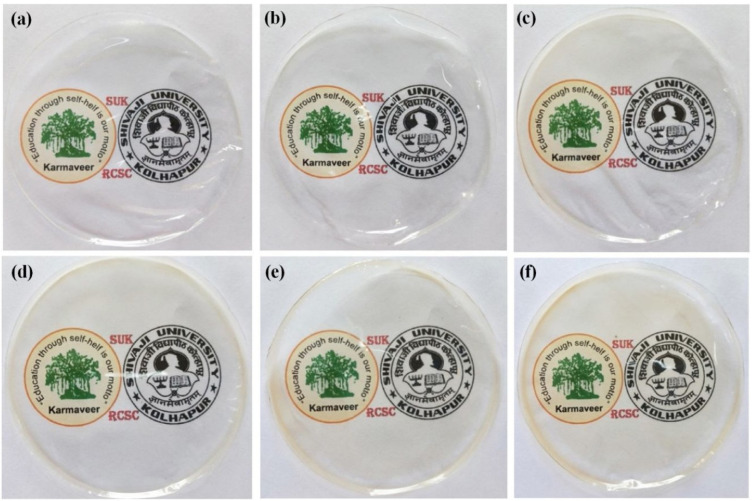
Photographic images showing the transparency of polyvinyl alcohol (PVA) film (**a**) and PVA/waste tea residue carbon dots composite films (**b**–**f**) with increasing concentration (0.5, 1.0, 2.0, 3.0, 5.0 mg) of waste tea residue carbon dots (taken from [6]).

**Figure 2:**
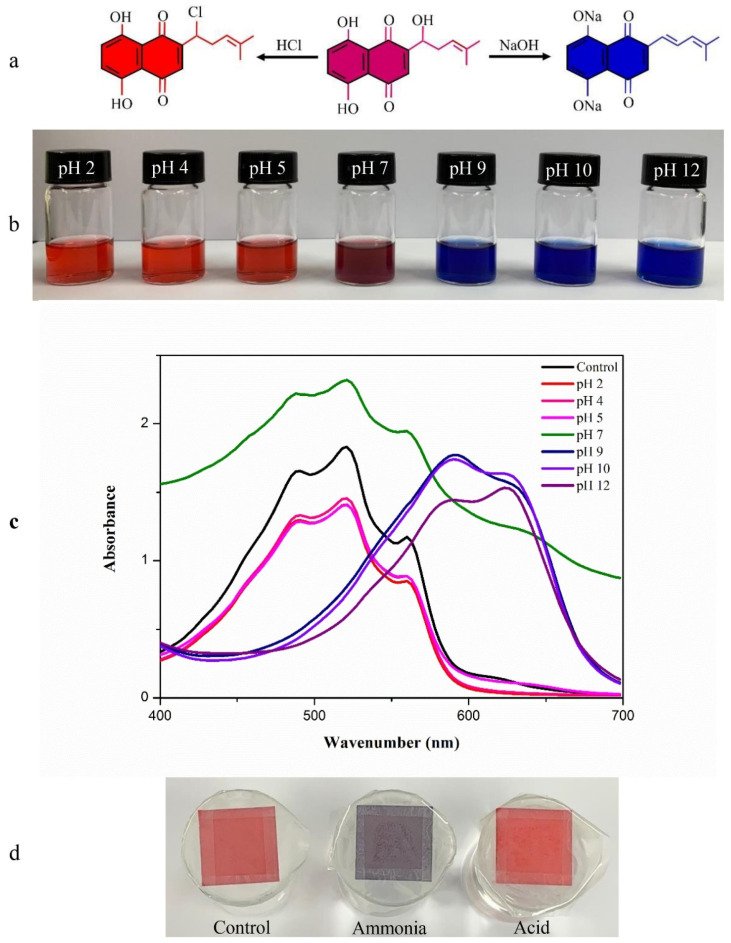
(**a**) The reaction of shikonin at acid-base conditions (**b**) color of shikonin solution at various pH levels, (**c**) UV–vis spectra of shikonin solution at various pH levels, and (**d**) response of the color indicator paper to ammonia and acetic acid vapors (taken from [7]).

In recent years, the field of food packaging has gained much importance. This is because consumers have become conscious about the quality of the food products they consume. Food products are generally classified either as fresh food or processed food. The employment of packaging technologies to food products ensures delivery in fresh or good conditions to the consumers [1]. This can be attributed to the fact that the packaging materials help to preserve the food products for a prolonged time. In other words, packaging improves the shelf life of food products. The increase in the shelf life of food products is of utmost importance, as food products are cultivated or processed in a place that is far away from the consumer. The transportation of food products to retail shops may take a long time, sometimes even spanning into months. It is important to note that food products may need to be stored at different locations during this transit period before being sold to consumers. Consumers also store food products for a definite period before consuming them. The packaging systems should prevent the spoilage of the food products until they are consumed [2]. Spoilage can be categorized as awfully hazardous, due to toxic microbes, or minute loss, due to changes in texture, loss of flavor, or color. Therefore, the material used for packing should hold antimicrobial characteristics that assist in the extension of the lag phase and simultaneous reduction of the growth phase of microbes [3]. This will be helpful in the extension of the shelf life of stored foods. In ancient times, food was stored in vessels or shells made up of leaves, woven grasses, bamboo logs, etc. [4]. Unfortunately, these packaging systems suffered several disadvantages, such as an unsophisticated appearance, transfer of odor from the storage vessel to the stored food, and shorter duration of storage [2,4]. These primitive packaging systems could not protect the food products from microbial contamination and spoilage by *Listeria monocytogenes*, *Micrococcus lutes*, and *Clostridium botulinum*, resulting in the food’s wastage products affecting the health of the consumers [5]. The reasons above necessitated the research on innovative food packaging systems, which can be made available to the food industry at economical prices. Further, current food packaging systems are expected to be biodegradable or edible [4]. Transparent packaging systems allow monitoring of the food quality without disturbing the external packaging (Figure 1). Of late, researchers are trying to develop smart packaging systems, which can divulge information about the packaged food products’ quality by changing the packaging system’s color (Figure 2).

Conventionally, petroleum-based polymers have been explored magnanimously to develop food packaging systems. Unfortunately, these packaging systems are not biodegradable. Due to their non-biodegradable nature, these systems exert a huge environmental burden. They have been identified as significant environmental hazards by the United States Environmental Protection Agency [4]. In this regard, polyhydroxyalkanoates (PHAs), a biodegradable alternative to petroleum-based polymers, have been recognized and used in food packaging. Although PHA holds a biodegradable nature, their biological and commercial production is limited and involves high costs [8]. Some other biodegradable polymers explored include alginate, gelatin, tamarind gum, chitosan and its derivatives, agar, etc. Among these polymers, chitosan and its derivatives have received much attention in the food packaging industry [1]. Besides the biodegradable nature of chitosan and its derivatives, they also possess antimicrobial and antioxidant properties [1]. The chitosan derivatives (cationic) have better antimicrobial actions against Gram-positive bacteria compare to Gram-negative bacteria or yeast [9]. The cationic charge of chitosan and its molecular weight firmly command its antimicrobial activity. This additional antimicrobial property allows researchers to develop functional films that can significantly enhance the shelf life of food products. The current review will discuss the properties of chitosan, methods for developing food packaging systems, and their applications in food product storage and safety.

## 2. Properties of Chitosan

**Figure 3:**
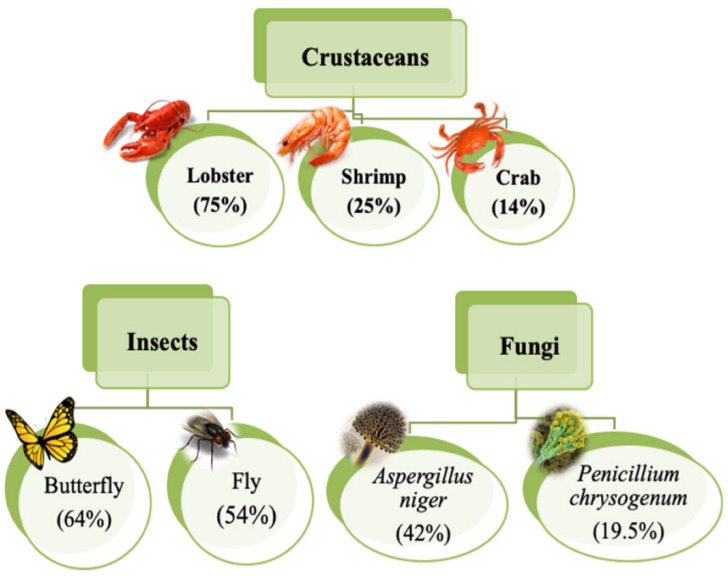
Different sources of chitin (taken from [13] under Creative Commons License).

**Figure 4:**
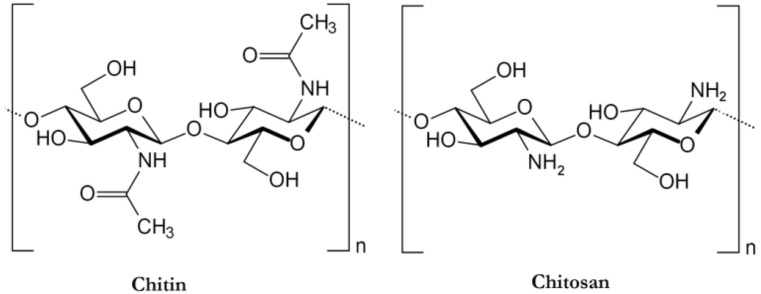
The chemical architecture of chitin and chitosan (taken from [11] under Creative Commons License).

**Figure 5:**
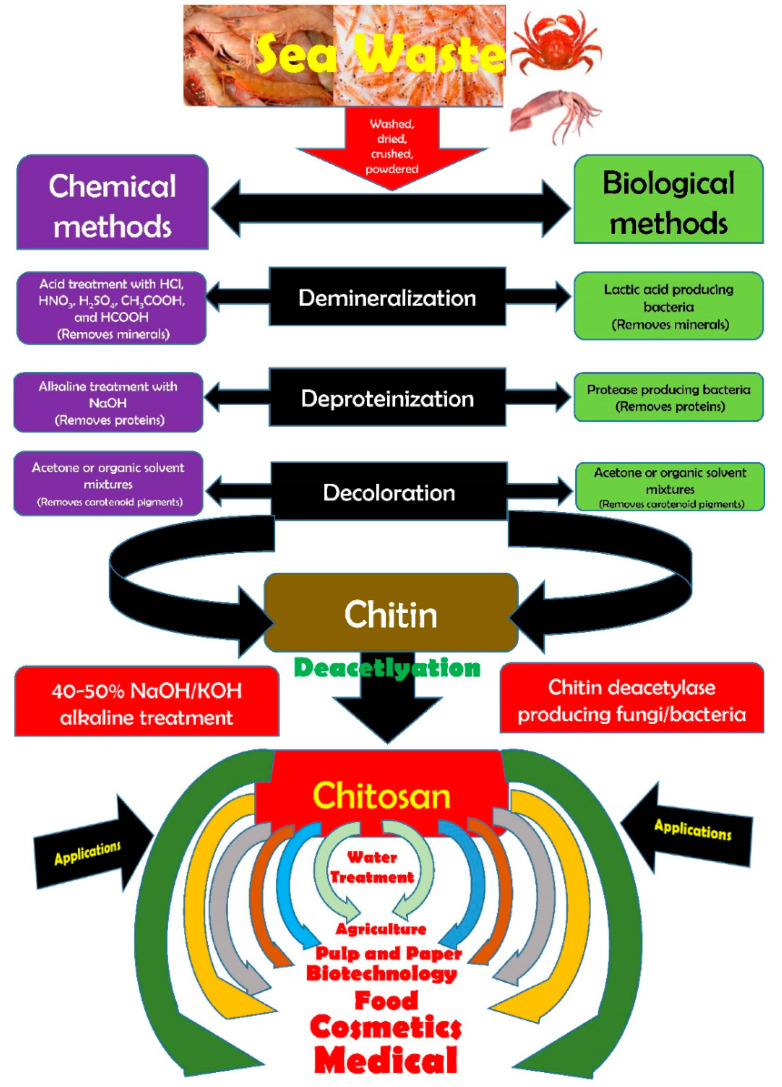
Modalities for the synthesis of chitosan and its applications (taken from [12] under Creative Commons License).

The food processing industries that deal with the different kinds of seafood generate many waste products, e.g., skins, shells, scales, internal tissues, etc. The weight of these wastes can be as high as >70% of the seafood source animal or organisms. The improper disposal (e.g., landfill, dumping in the sea/local water bodies, or burning) of these wastes can lead to increased environmental hazards. Hence, researchers have proposed the valorization of the waste products to the extent possible, which can reduce the burden on the environment to a great extent. The food industries dealing with crustaceans have generated ~75% of waste products [4]. The waste products of the crustacean industry are rich in proteins and chitin (a naturally occurring polysaccharide). The polysaccharide can also be extracted from the biomass of fungi, fly larvae, annelids, and yeasts (Figure 3) [1,4,10]. The natural abundance of chitin is second-most, after the cellulose. However, chitin is the most abundant polymer of natural origin [4]. Chitin is an amino polysaccharide. Chemically, chitin is composed of β-1,4 linked 2-(acetylamino)-2-deoxy-d-glucose elements (Figure 4) [4,10,11]. The degree of acetylation is >90%. The overall nitrogen content in chitin is ~7%, while the nitrogen to carbon (N/C) ratio is 0.146. This naturally occurring polymer is biodegradable, biocompatible, and has been explored for several food applications [10]. Unlike other polysaccharides (e.g., alginate, tamarind gum, guar gum, etc.), chitin is insoluble in traditional solvents, limiting its applications [10]. Due to this reason, researchers have proposed converting chitin to chitosan (Figure 5). This can be achieved by the controlled deacetylation of chitin [1]. The deacetylation of chitin is mainly carried out by two methods: the alkaline method and the enzymatic method. The alkaline method of deacetylation is carried out by refluxing chitin in 40–50% of sodium hydroxide solution for a specific period [12]. Unfortunately, this method is not environmentally friendly. The enzymatic method involves treating the chitin with a chitin deacetylase enzyme extracted from various bacterial strains, which is preferred to deacetylation, due to its eco-friendly nature [12]. Inside the chitin backbone, the deacetylation partially eliminates the acetyl groups from the acetylamino groups. This results in the conversion of the constituent elements of chitin to β-1,4 linked 2-(amino)-2-deoxy-d-glucose elements (Figure 4) [4,11]. The ^1^C_4_ conformation of the linkages of monomer units, formed due to equatorial glycosidic linkages, restricts the movement of the monomers to a great extent. This phenomenon makes the chitosan a stiff molecule, explaining the high intrinsic viscosity of chitosan solutions [4]. The free amino groups are mainly responsible for the chemical and functional properties of chitosan. Typically, the degree of acetylation in chitosan is lower than 40%. The deacetylation process does not eliminate the nitrogen from the polysaccharide. Hence, the nitrogen content of chitosan is always >7% [4].

**Figure 6:**
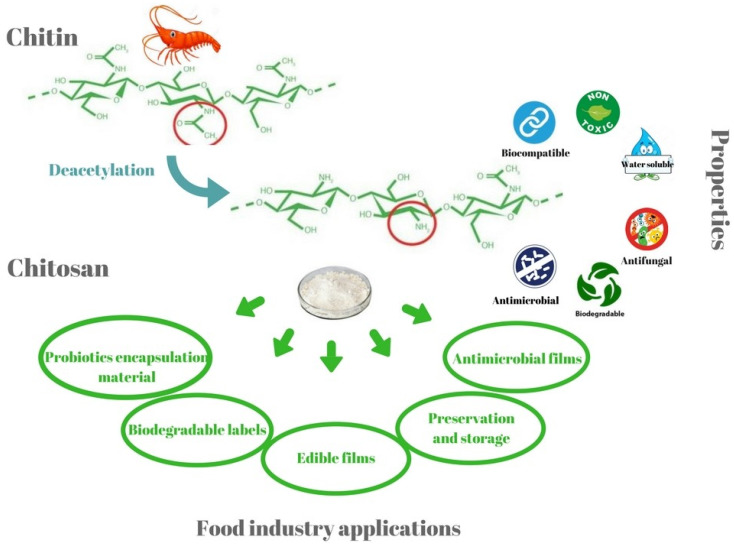
Some of the unique properties and food industry applications of chitosan (taken [15] under Creative Commons License).

The solubility of chitosan in water is a major challenge for food scientists. It is important to note that the presence of a free amino group makes chitosan a basic polysaccharide. Due to this reason, the chitosan is solubilized at low pH solutions (usually below the pH of 6) [4]. However, some chitosans with a comparatively higher degree of acetylation (50%) solubilize at a pH of 7 [4]. Usually, chitosan has been reported to be solubilized in acidic solutions (e.g., hydrochloric acid, lactic acid, and acetic acid, etc.). Chitosan can be solubilized in weak acids, such as lactic acid and acetic acid, for food applications. Further, few researchers have reported converting chitosans into salts (e.g., chitosan lactate and chitosan glutamate) and derivatives (e.g., carboxymethyl chitosan). The conversion of chitosan into chitosan oligosaccharide, a low molecular weight chitosan product, which is soluble in water, has also been proposed. Chitosan and its derivatives have received much attention in the food industry, mainly for its antimicrobial and antioxidant properties (Figure 5). The polysaccharide has also been explored due to its viscosity-enhancing properties as a stabilizing agent, emulsifier, and controlled flavor-release matrices. The chitosan matrices have also been used to immobilize various food and nutraceutical agents, such as enzymes, vitamins, probiotics, etc. (Figure 6). Chitosan matrices have been used to immobilize the methanotrophs that resulted in improvised methanol production [14]. Since chitosan is a bulk-forming agent, it also serves as a source of dietary fibers for improving gut health.

**Figure 7:**
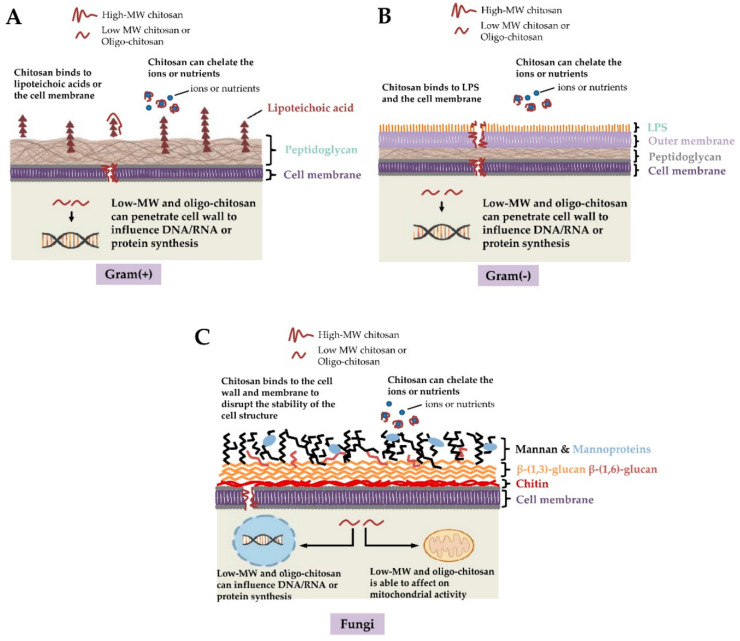
Mechanism of antimicrobial property exhibited by chitosan (taken [16] under Creative Commons License).

As reported in the previous paragraph, chitosan exhibits antimicrobial properties. This property allows the polymer to be used as a natural antimicrobial agent/preservative. Hence, the packaging materials developed with chitosan and its derivatives inherently exhibit antimicrobial properties towards a wide range of bacteria, fungus, and viruses. It is important to note that chitosan is biologically active against many microorganisms, including pathogenic microbes. Chitosan-based packaging systems have been found to prevent food products from Gram-positive and Gram-negative bacterial growth and fungal growth (Figure 7) [1]. The antimicrobial activity of chitosan has been related to the free amino group of the glucosamine residue. This fraction of chitosan can be easily protonated. The protonated amino groups then interact with the cell membranes of the microbes that are inherently anionic. The anionic behavior of the microbes can be explained due to the presence of phospholipids, lipopolysaccharides, peptides, and amino acids present on the surface of the microbial cell membranes [1]. The interaction between the cationic chitosan, or chitosan derivatives, and the anionic microbes, increases the permeability of the cell membranes and can disrupt the cell membranes. This results in the leakage of the internal cellular matter leading to the death of the microbes. Another plausible mechanism by which chitosan is expected to inhibit microbial cellular growth is the interaction of the chitosan molecules with the microbial cells’ genetic material (DNA or RNA). This inhibits DNA transcription, RNA translation, and protein synthesis [1]. A group of researchers also suggested that since chitosan is a cationic polyelectrolyte, it acts as a chelating agent for various ions, such as Cu^2+^, Hg^2+^, Zn^2+^, Cd^2+^, and Ni^2+^ [4]. Due to this reason, chitosan can interact with the essential nutrients and trace elements that are required for cell growth and toxin production by the pathogenic microbes, respectively [1]. Additionally, the chitosan molecules can also interact with the spore of the microbes and consequently neutralize them, which helps to prevent the growth of the microbes [1].

**Figure 8:**
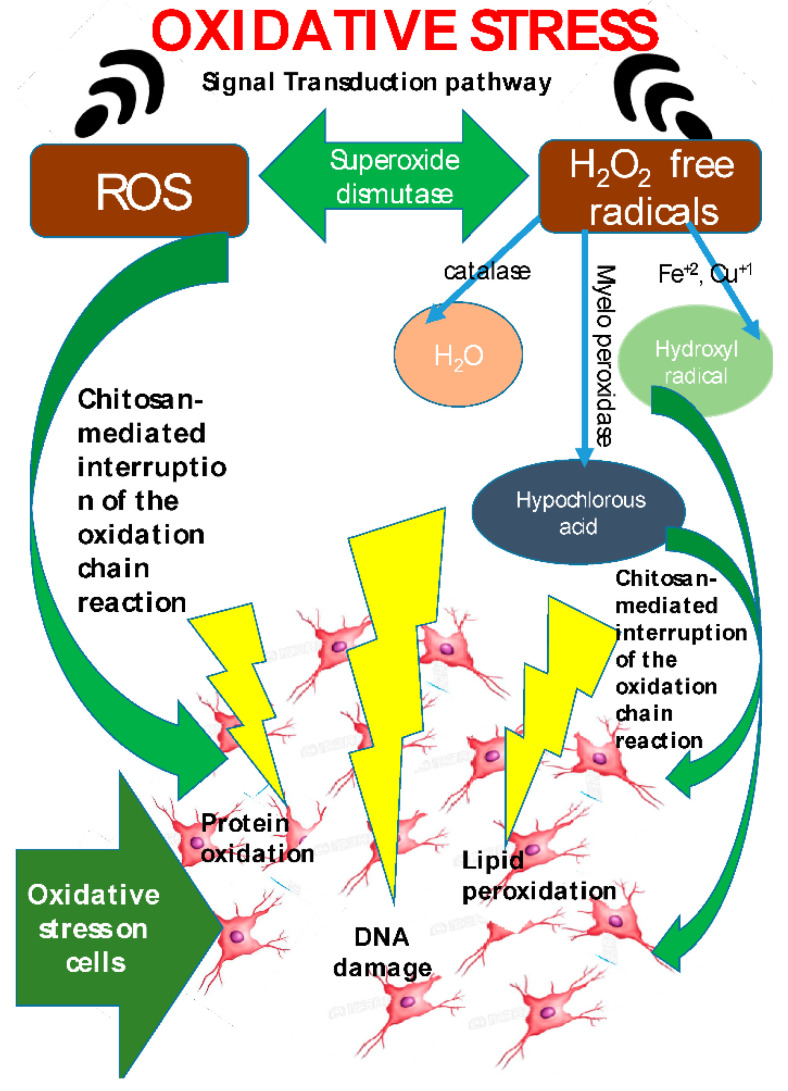
Mechanism of anti-oxidative property exhibited by chitosan (taken from [12] under Creative Commons License).

The antioxidant property of the chitosan and its derivatives have been related to the free radical scavenging activity of the chitosan (Figure 8). Chitosan derives this property from the lone pair of electrons on the nitrogen atom located at the C-2 position. The lone pair of electrons allows the nitrogen atoms to accept a proton to form an ammonium (NH_3_^+^) group, which immediately reacts with the free radicals to generate stable molecules [17]. The antioxidant properties of chitosan and its derivatives have been extensively studied and documented using the 2,2-Diphenyl-1-picrylhydrazyl (DPPH) free radical and 2,2′-azino-bis(3-ethylbenzothiazoline-6-sulphonic acid) (ABTS) assays [18].

## 3. Methods for the Development of Food Packaging Systems

Food scientists and researchers working in the food packaging industries are looking forward to developing active packaging films using bio-derived polymers. This is because conventional packaging materials are non-biodegradable. On the other hand, bio-derived polymers are bio-degradable polymers. Among the several bio-derived polymers, chitosan is one of the most sought-after polymers, which is regarded as an “active” polymer due to its inherent antioxidant and antimicrobial properties. The polymer is non-toxic and biocompatible. It does not intrinsically contaminate food products and has been regarded as safe for human consumption by the US-FDA. Hence, chitosan is being explored as a suitable alternative to conventional plastic-based materials, which are non-biodegradable, for developing food packaging systems. Since the polymer is obtained from the by-products of the other food industries, it creates an opportunity to design food packaging systems economically. Chitosan has a facile film-forming capability similar to many other polysaccharides (e.g., tamarind gum, guar gum, and alginic acid). The films of chitosan are biodegradable and exhibit good mechanical properties. The mechanical and other properties of the chitosan films can further be improved by employing different polymer processing technologies, such as polymer blending and reinforcement with nanoparticles. Additionally, the chitosan films have shown good water vapor and oxygen barrier capacity, which supports the candidature of the chitosan to be used as a food packaging material. In this current section, an attempt will be made to discuss the different methodologies that have been employed to develop chitosan-based food packaging systems.

### 3.1. Solution Casting Method

**Figure 9:**
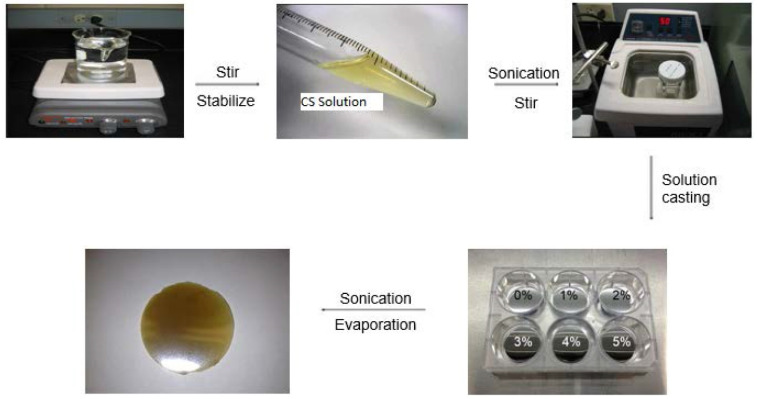
A generalized method for the synthesis of chitosan films (taken from [19] under Creative Commons License).

The solution casting method, also known as the direct casting method, is one of the most commonly used to prepare polymeric films for food, pharmaceuticals, and biomedical applications (Figure 9). This method is straightforward and has been used for a long time to develop film-based polymeric systems. Hence, many authors regard this method as the conventional solution casting method. The method involves the dissolution of the polymers in suitable solvents. The polymeric solutions, which may contain various additives, such as plasticizers and crosslinkers (e.g., genipin and glutaraldehyde), are degassed. The degassing can be achieved either by applying a vacuum at a slightly elevated temperature or by ultrasonication. Then the degassed solutions are poured into molds, which are placed on a leveled surface, and subsequently dried under a controlled environment of a specific temperature and humidity. The molds are usually made up of non-sticky materials (e.g., Teflon) such that, after drying, the films can be easily peeled off from the molds. After that, the peeled-off films are cured, if needed, and stored for further use. Since this method’s preparation is relatively easy, many researchers have extensively used it to develop films as food packaging material with significant efficacy. Though the method has shown great promise in the food packaging industry, utmost care must be taken while designing films by this method. A slight deviation in the process parameters may result in batch-to-batch variations in the properties of the films [4].

The blends of chitosan can also be used to develop films. Blends are prepared by mixing two or more polymers to tailor the physical properties of the resultant films over the films made with pristine polymer(s). The polymeric blends may either form a homogenous matrix or a phase-separated matrix system. It has been reported that blends of chitosan with different polymers (natural or synthetic) can improve the food packaging materials’ physical, mechanical, thermal, optical, and barrier properties [1]. The natural polymers used for this purpose are classified as polysaccharides and proteins [1]. The derivatives of these polymers have also been employed. The examples of polysaccharide-based natural polymers include pectin, starches from different sources (e.g., cassava starch, purple yam starch, and potato starch), bacterial cellulose, carboxymethyl cellulose, and gum arabic [1]. Protein-based natural polymers include collagen, gelatin, fish myofibrillar protein, soy protein isolate, and zein [1]. Some of the synthetic polymers employed in conjunction with chitosan include poly (vinyl alcohol), poly (ε-caprolactone), and ethylene-vinyl alcohol [1]. These polymers are mixed with chitosan solution and homogenized before the degassing stage, and this is then used for the film casting.

**Figure 10:**
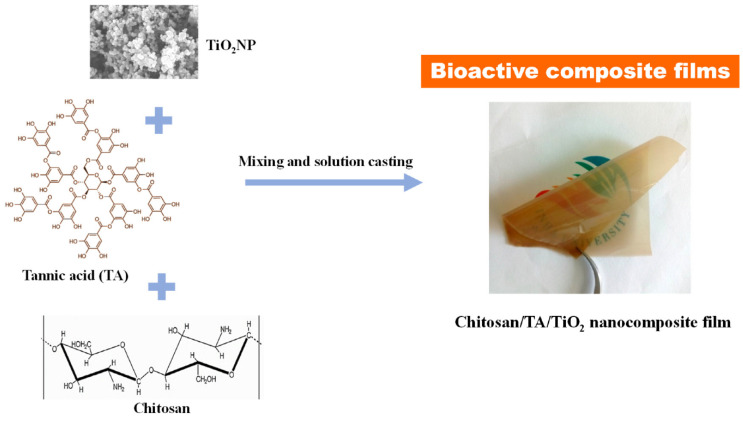
Representative process for the development of nanocomposite films (taken from [20] under Creative Commons License).

The functionality of the chitosan films (pristine or blend) can further be improved by adding additives. Various additives, such as essential oils and nanoparticles, are being added to enhance the films’ antimicrobial, antioxidant, and other properties (e.g., mechanical and barrier). These components are added to the chitosan solution or chitosan blends and are subsequently homogenized to form stable emulsions or dispersions. These emulsions or dispersions are then degassed before casting into films (Figure 10).

### 3.2. Coating of Food Products

**Figure 11:**
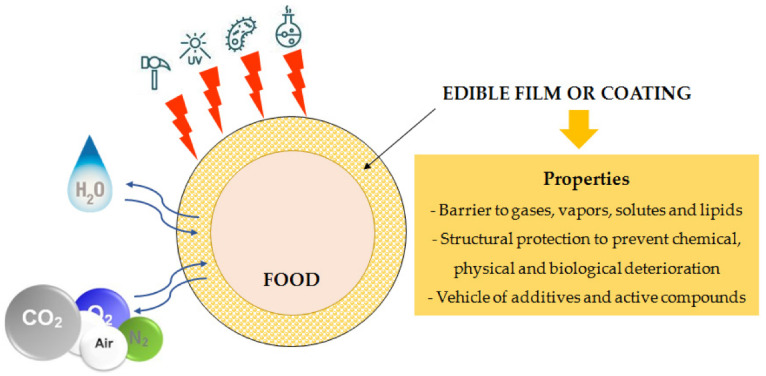
Schematic representation of the advantages of edible food coatings (taken from [22] under Creative Commons License).

The coating of food products involves creating a polymeric film layer directly on the surface of the food products. These polymeric layers are expected to delay the degradation of the food products, which would allow for the extension of the shelf life of the food products. The shelf life extension can be explained by the barrier effect exerted by the coatings (Figure 11). Due to the barrier effect, the layers can prevent moisture loss, promote the exchange of gases, and prevent microbial growth over food products. These factors help to delay the degradation of food products [21]. The coatings over the food products can be achieved by spread coating, spray coating, or dip coatings methods [4]. In this section, all three methods will be discussed in detail. 

#### 3.2.1. Spread Coating

**Figure 12:**
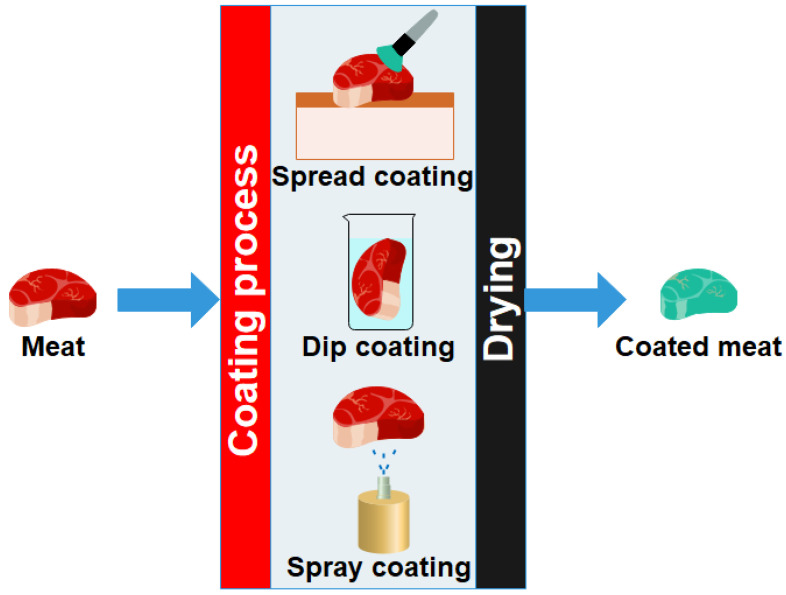
Schematic representation of spread coating, dip coating, and spray coating.

The spread coating method is the easiest method of polymeric coating over the surface of food products and packaging materials (Figure 12). There is no requirement for employing a sophisticated instrument. Due to this reason, this method can be easily adapted by small-scale industries without much financial burden. In this method, the polymer solutions are directly applied over the food product or packaging materials using a sterile brush or spatula [4]. The polymer solution may contain several additives (e.g., essential oils, nanomaterials, and plasticizers, etc.) that would improve the physical, barrier, and mechanical properties and improve the coatings’ functionality (e.g., antimicrobial and antioxidant). Once the polymer solution/mixture is coated over the food products, the food products are dried under sterile conditions. For this purpose, a laminar flow cabinet can be used [4]. After the liquid coating layer is dried to form a thin film, the food products are subsequently packaged and stored under the specified storage conditions for the food products.

The coating of the packaging materials that do not have functional properties, such as antimicrobial and antioxidant properties, can be carried out similarly. The coating imparts additional available properties to the packaging materials. For example, coating the paper-based packaging material with chitosan coating imparts antimicrobial and antioxidant properties to the packaging system [4]. Further, such a coating system improves moisture retention without compromising the mechanical properties [4].

#### 3.2.2. Dip Coating

Similar to spread coating, dip coating is also widely used and can be easily implemented (Figure 12). In this method, the food products are dipped into the coating solution and housed for some time. The dipping of the food products is usually carried out at a specific speed. The speed of dipping should be optimized for a food product-coating solution pair. The dipping speed has been found to affect the coating performance. This step allows the deposition of the coating materials over the food surface to take place. The vegetables and fruit are usually immersed in the polymeric solution for 5–30 s [23]. After that, the solvent is evaporated, resulting in the formation of a thin film. The viscosity of the polymer solution governs the thickness of the coating layer. In general, higher viscosity of the polymer solution/dispersion results in a thicker coating layer. The formation of a thick coating layer may interfere with the respiration process and reduce the shelf life of the food products. Further, even a slight variation in the process parameter can significantly affect the quality of the coating layer.

#### 3.2.3. Spray Coating

Spray coating is the most commonly used method for coating food products with a high extent of repeatability (Figure 12). In the spray coating method, the coating liquid is converted into fine droplets, which are then sprayed over the food products. The fine droplets form a thin liquid film over the food surface. The liquid films are then dried to form a thin solid film over the food surface. Food industries commonly utilize spraying techniques, such as air spray atomization, air-assisted airless atomization, and pressure atomization. The air spray atomization-based sprayer uses a high velocity of air for the atomization process. This method of spray coating is the most economical way to coat food products. However, the process fails to handle high-viscosity liquids [23]. The air-assisted airless atomization-based sprayer is a two-stage atomization unit. In the first stage, partial atomization of the polymer solution is achieved. In the second stage, compressed air is used for complete atomization. This type of sprayer is capable of handling high viscosity liquids and high-solid coatings. Further, this technique provides a high throughput output with high-quality finishing of the coated surface [23]. The pressure atomization-based sprayers use pressure, which can be raised to 3.5 bars. This type of sprayer works without air and can handle high viscosity liquids [23].

### 3.3. Extrusion Method

**Figure 13:**
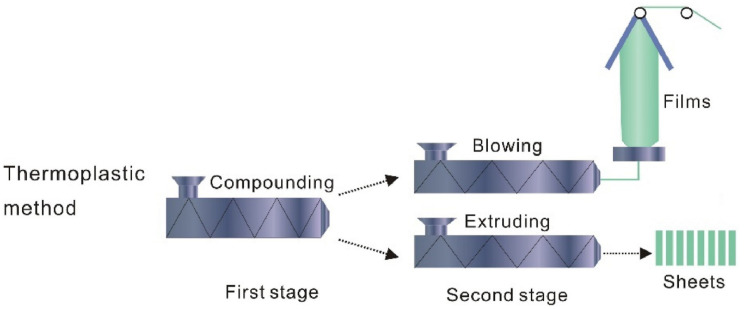
Schematic of film production by extrusion method (taken from [24]).

The extrusion method is also used for the development of films for food packaging applications. Melt extrusion is one of the commercial methods that is widely explored by the food industry for developing food packaging systems. This method does not use any solvents. Hence, the extrusion method is also regarded as a dry process. A typical extruder machine has three components: feeder zone, kneading zone, and extrusion zone [23]. The extrusion zone is attached to a heater to heat the material feed, which helps to extrude flat films. A blown film extruder can be used to prepare films (Figure 13) [4]. Chitosan-based active packaging systems have also been developed by the extrusion method. The films produced by the extrusion method have superior mechanical properties. Plasticizers (e.g., glycerol and polyethylene glycol) can be added to increase the ductility of the films [23].

### 3.4. Multi-Layered Films

In many cases, multi-layer packaging materials have been proposed. These packaging materials employ more than one polymer, which is similar to polymer blends. Therefore, these systems benefit from the valuable properties of the constituent polymers. However, in the multi-layer systems, initially, a film of the first polymer is developed. Over the first layer, another layer of polymer is deposited, and so on. Due to this reason, each of the layers retains its properties. Hence, the resultant films have properties of each constituent layer, unlike the polymer blends, where the properties are tailored based on the ratio of the mixture solutions used to develop the films. The main advantage of this method is that the existing techniques can be employed to fabricate the films. Further, it is possible to combine any of the methods above to develop multi-layer films.

### 3.5. Flexographic and Screen Printing

**Figure 14:**
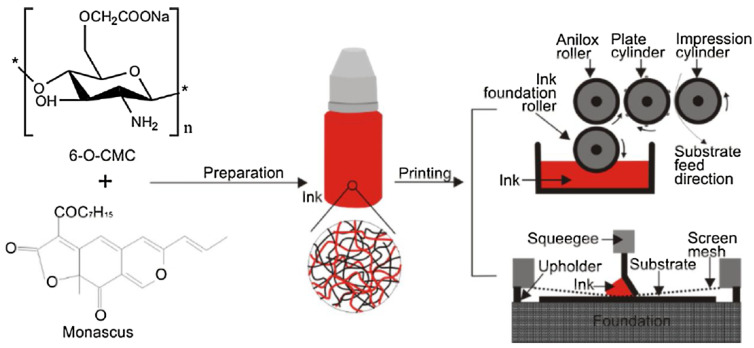
The preparation and printing of carboxymethyl chitosan-monascus based inks (taken from [25]).

In recent years, edible inks have received significant attention from food scientists across the globe. This can be ascribed to the environmentally friendly nature of the edible inks. Further, edible inks are categorized as safe for human consumption. The use of edible-grade resin (e.g., chitosan and its derivatives) and food-grade colorants (e.g., monascus pigments) can explain this. The food colorants provide desirable colors to the films formed by edible films. Depending on the application of the edible inks, they may contain flavoring agents or taste-enhancing agents. Further, the edible inks may be loaded with antimicrobial agents too. The edible inks may be flexographic or screen printed on the substrates, either on the primary packaging material or the food products. The coating process has been summarized in Figure 14.

### 3.6. Electrospinning

**Figure 15:**
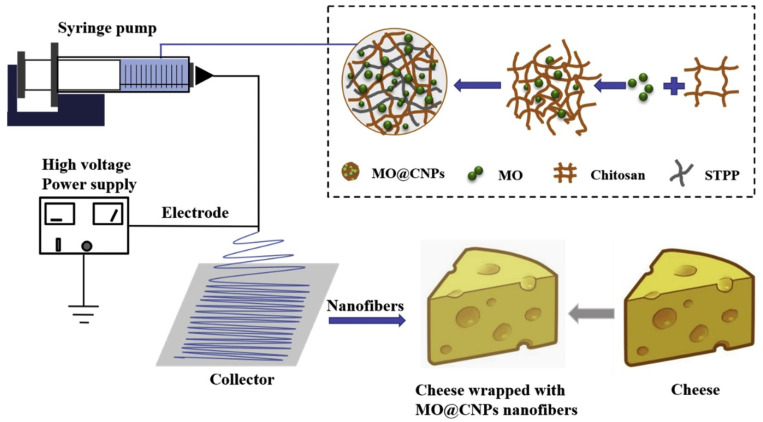
Schematic of conventional electrospinning method for developing films (taken from [27]).

**Figure 16:**
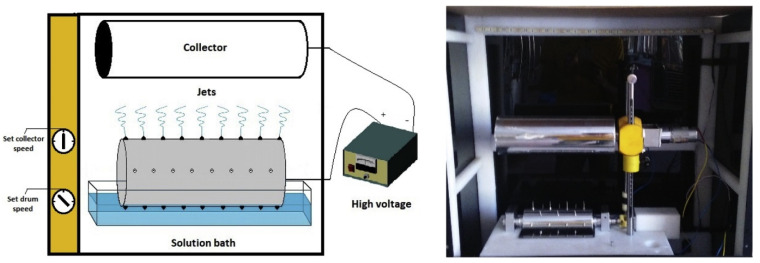
Schematic and image of nozzle-less electrospinning device (taken from [28]).

Food researchers widely study the electrospinning technique to encapsulate bioactive compounds within polymeric nano-fibers. The electrospinning technique is the simplest method to develop nano-fibers, which typically have fiber diameters in the range of 5 nm to 10 μm. The thickness of the fiber diameters can be altered by changing the composition of the polymer solutions. In general, higher polymer concentration leads to the formation of thicker fibers. In many cases, several polymer solutions are blended to obtain nano-fibers of specific characteristics. This method can be easily tailored to develop films for food packaging applications. In this regard, edible polymers, including chitosan and blends of chitosan, have also been explored to develop films for food packaging applications [26]. The conventional electrospinning technique uses a nozzle-based system for the development of the films. However, the growth of films by this method is quite slow. Hence, commercial establishments usually employ a nozzle-free system for the preparation of the films. The nozzle-free system can generate a high volume of nano-fibers and can therefore develop the films very quickly. Though there is a significant difference in the rate of formation of nano-fibers among both the electrospinning systems, both the systems employ a very high voltage to develop nano-fibers. Figure 15 and Figure 16 provide the representation of the nozzle-based and nozzle-free electrospinning systems for the development of the films.

## 4. Applications of Chitosan-Based Films

### 4.1. Chitosan Films Containing Natural Compounds and Essential Oil

**Figure 17:**
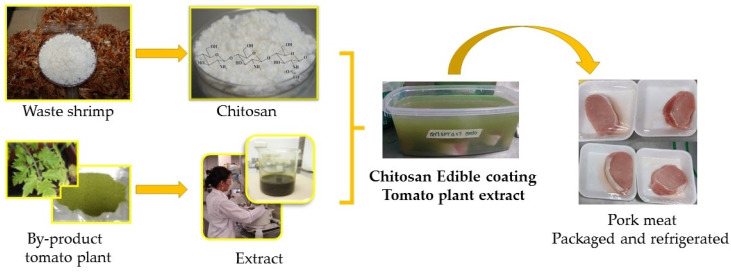
Schematic representation of the development of natural extract-containing edible food coatings (taken from [33] under Creative Commons License).

**Figure 18:**
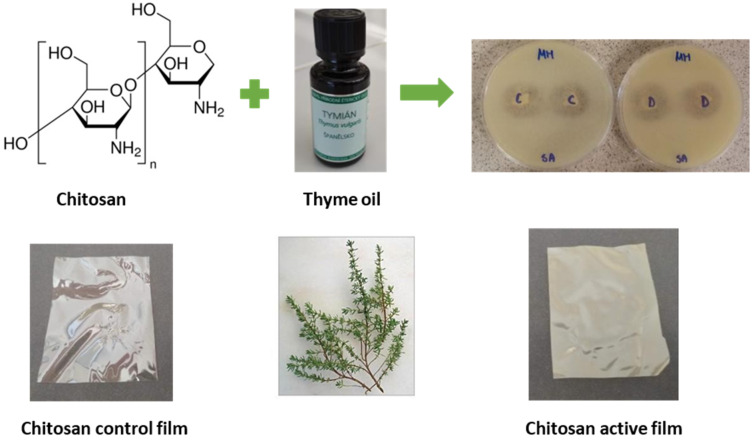
Schematic representation of the development of natural extract-containing edible food coatings (taken from [34] under Creative Commons License).

The proper packaging of fresh and processed food products helps distribute, store efficiently, and market the finished products. Various researchers have used different types of natural polymers to develop packaging materials. Chitosan is one such polymer. In [29], the researchers have explored the ability of the high, medium, and low molecular weight of chitosan as the packaging material. In the study, the authors reported the inherent antimicrobial and antioxidant properties of the chitosan films. The films made with high molecular weight chitosan provided better results than the low and medium molecular weight films. The optimized film was used as a packing material for the preservation of strawberries. It was found that the chitosan film performed better than the commercially available polyethylene films in maintaining the physical and chemical properties of the strawberries [29]. However, active food packaging systems consist of various functional additives that can enrich the functional properties of the chitosan films. In other words, though chitosan exhibits different inherent properties, such as antimicrobial, antioxidant, and UV-blocking properties, these functional activities can be further improved by adding additives with active properties. It has been reported that chitosan films’ antioxidant and antimicrobial properties are relatively low [30]. To outmaneuver this shortcoming, various researchers have proposed to add different types of antioxidants and antimicrobials within the chitosan films. The addition of various natural additives is preferred over synthetic additives. This is owing to the toxicological effects exerted by the synthetic additives [30]. Different researchers have proposed using plant extracts (e.g., *Artemisia campestris* extracts, peanut extracts, and grape seed extract) and essential oils (e.g., citronella essential oil, bergamot essential oil, and cedarwood essential oil). This substantiates the inherent properties of the chitosan films (Figure 17 and Figure 18). Further, the conversion of chitosan into quaternary ammonium chitosan has also been proposed to improve the antimicrobial activity of chitosan [31]. This is because the quaternary ammonium compounds can quickly kill the bacteria when brought into contact with them. The improvement in chitosan films’ antibacterial and antioxidant properties is achieved by adding polypyrrole [32]. However, in this section, the packaging films of chitosan consisting of natural compounds and essential oils will be discussed further.

The chitosan films containing the extracts and essential oil of *Artemisia campestris* were developed with improved antioxidant and UV and visible light-blocking properties. The authors reported that the hydroethanolic extracts and the essential oil of *Artemisia campestris* increased the films’ water resistance and thermal stability by up to 300 °C. [30]. The hydroethanolic extracts of *Artemisia campestris* increased the hydrogen bonding within the chitosan films. The UV and visible light blocking properties are of paramount consideration. This is because the exposure of food products to UV and visible light has been proven to degrade many food products via the oxidation process. The additive-loaded films were found to inhibit oxidation by the direct intervention of polyphenols (known antioxidants), and UV and visible light-blocking properties. These films were found to be acceptable to be explored as active food packaging films. A similar study with the root extracts of Chinese chives (Allium tuberosum) was also performed in [35]. The extract consists of a large number of phenolic compounds. Some of the major phenolic compounds in Chinese chives include gallic acid, isoquercetin, catechin, ferulic acid, p-coumaric acid, and rutin [35]. The antimicrobial activity of the chive extract can be explained by the presence of allyl methyl sulfide and diallyl disulfide. The chive extract-loaded chitosan films exhibited good biodegradability and improved antioxidant and antimicrobial properties over the control film [35]. Like gallic acid, boswellic acid has also been exploited as an active agent in food packaging applications. Chemically, boswellic acid is a pentacyclic triterpenic acid, which is extracted from the *Boswellia serrata* tree. The inclusion of the boswellic acid helped to improve the UV-blocking property of the chitosan/PVA blend films. The said active agent improved the mechanical property of the films but did not deteriorate the thermal property of the films. The films were able to prevent the proliferation of bacterias, such as *E. coli*, *S. aureus*, and *C. albicans* [36]. Similar observations were also made with rutin-incorporated chitosan/PVA blend films [37]. Another group of researchers has explored the extracts of pine needles (*Cedrus deodara*), which consists of curcuminoid pigments, as a functionally active agent for synthesizing chitosan-based packaging materials. Curcuminoids are composed of polyphenolic compounds, having excellent antimicrobial and antioxidant properties. The addition of pine needle extract within the chitosan films increases the barrier properties of chitosan films [38]. The antioxidant properties of the pine needle extract-containing films improved significantly. In [39], grapefruit seed extract was loaded with caprolactone/chitosan films. The films had sufficient mechanical properties and flexibility to be explored as active food packaging material. The antibacterial activity of the films against *E. coli* and *P. aeruginosa* suggested that the growth of the microbes could be inhibited for 120 h. The packaging application of the developed films was evaluated on salmon and bread under storage conditions. It was found that the proposed films enhanced the shelf life of salmon and bread by inhibiting *E. coli* and mold, respectively [39].

The addition of natural colorants, obtained from different sources of plants, for the synthesis of food packaging films, has been proposed by various researchers. Apart from imparting color to the films, the addition of these colorants serves multiple purposes that can make the films multifunctional. One such example of natural colorant is betalains, which is a mixture of nitrogenous compounds, namely betacyanins and betaxanthins. It is generally extracted from plants from the families of *Amaranthaceae*, *Cactaceae*, and *Chenopodiaceae*. However, it can also be removed from other sources, such as beetroot and red pitaya [40]. The incorporation of betalains within the chitosan/gelatin films was found to increase the mechanical properties of the films. The improvement in the mechanical properties was due to the increment in hydrogen bonding. The ductility of the films also increased significantly. The betalains-loaded films significantly improved the UV-visible light-blocking and antioxidant properties. It also improved antimicrobial properties against several foodborne pathogens (e.g., *E. coli*, *S. aureus*, *S. typhimurium* and *L. monocytogenes*) [40]. The betalains-rich vegetable amaranth extract has been found to elicit color change when the pH of the solution is changed. At pHs until 9.00, it exhibits different shades of red, while at pH 10.00, the color changes to green, and at subsequent higher pHs, the color becomes yellow. Incorporating such color pigments within the packaging films makes them environment-sensitive, wherein the color of the packaging material changes if the pH of the food product increases considerably. Such packaging films are regarded as smart packaging films, which can provide information on the spoilage of the food products without opening the packaging [40].

The biodegradable antimicrobial active food packaging films were prepared using blends of chitosan and poly (vinyl alcohol) [41]. The films were developed by the conventional solution casting method and were found to be transparent. Natural extracts of *Ocimum tenuiflorum* were added to the films to substantiate chitosan’s antimicrobial activity within the films. It was observed that the addition of the natural extracts significantly affected the water-resistance of the films. The DPPH radical scavenging experiment assessed the antioxidant property of the films. The antioxidant value of the film was found to be ~41%. The prepared films were reported to help enhance food security. The mechanical property of the chitosan/PVA blends improves by functionalizing chitosan with catechol. Such films were found to have better antibacterial and UV-blocking properties [42]. In [43], the protein hydrolysates from shrimp and crab protein have been explored as additives to develop films of chitosan and gelatin. The enzymatic hydrolysis of the proteins releases the bioactive peptides, eliciting beneficial properties to the edible films. Protein hydrolysates increase hydrophilicity, antioxidant and antimicrobial properties. The antioxidant properties of the films were estimated by the DPPH radical scavenging, ferric reducing power, and metal-chelating effect experiments. The antimicrobial activity was tested against Gram-positive (*Bacillus cereus*, *Staphylococcus aureus,* and *Micrococcus luteus*) and Gram-negative (*Escherichia coli*, *Klebsiella pneumoniae*, *Salmonella enterica*, *Salmonella typhimurium*, and *Enterobacter sp.*) bacteria. In a separate study, 3-Phenylacetic acid, a naturally occurring antimicrobial agent, has been explored to increase the activity of gelatin/chitosan films [26]. The antimicrobial agent is generally found in honey, fermented food, and many lactic acid bacterial species. It has a broad range of antimicrobial activities and is active against bacteria and fungi. The films had antimicrobial activity for *Salmonella enterica* and *Staphylococcus aureus*. The improvements in the mechanical and thermal properties of chitosan/guar gum/PVA blend films have been proposed by crosslinking the polymer matrix using hydroxy citric acid [44]. The water vapor permeability of the blend films was also curtailed post crosslinking. The prepared films were active against *S. aureus* and *E. coli* bacteria.

In recent years, photodynamic inactivation technology has been proposed. Herein, light-emitting diodes that generate photons in the visible region are used to sterilize the food products. When irradiated on the food products containing photosensitizers, the light in the visible wavelength generates in situ reactive oxygen species (e.g., singlet oxygen and hydroxyl radicals). Singlet oxygen is a strong antimicrobial agent and is capable of efficiently killing viruses and bacterias. Films with photodynamic inactivation activity are prepared by adding the photosensitizers within the polymeric solutions. One such photosensitizer is riboflavin, a water-soluble vitamin. The addition of riboflavin imparts a yellow color to the film. Additionally, riboflavin imparts UV and visible light barrier properties to the films. This, in turn, retards the photooxidation of the food products, thereby extending their shelf life. In [45], the authors reported the development of biodegradable riboflavin-loaded chitosan films. These films showed excellent antimicrobial properties when irradiated with UV light. It was found that the films could exterminate both pathogenic (*Listeria monocytogenes* and *Vibrio parahaemolyticus*) and spoilage bacteria (*Shewanella baltica*). The films were tested as a packaging material for salmon and were reported to be greatly effective in extending the shelf life of refrigerated food products.

**Figure 19:**
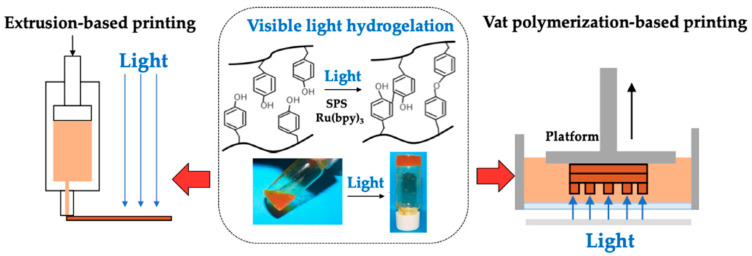
Schematic representation of the coating process using chitosan-based ink (taken from [47] under Creative Commons License).

Chitosan-based printing inks have been proposed in [46]. In the study, the authors analyzed the properties of the inks that were prepared using chitosan of different molecular weights. The inks were found to be suitable for printing applications. However, the solubility of chitosan in acidic solutions is a major disadvantage of such inks. It has been reported that the presence of acids can change the flavor and taste of food products and induce the corrosion of the printing machines [25]. Accordingly, edible inks of water-soluble carboxymethyl chitosan have been proposed to overcome this disadvantage [25]. The carboxymethyl chitosan-based edible inks were found to have good antimicrobial properties against *E. coli* and *S. aureus*. The authors concluded that the prepared inks could be explored for food packaging applications. Figure 19 represents the generalized coating processes using chitosan-based ink [47].

### 4.2. Chitosan-Based Nanocomposite Films

The functionality of the chitosan-based films can be improved by developing nanocomposite films of chitosan. It has been reported that the addition of nanoparticulate systems improves the physical and mechanical properties, temperature, and humidity resistance of the chitosan-based films, and can increase the antimicrobial activity of the films. In this regard, various nanoparticles (e.g., cellulose nanocrystals, nanosilver, nano-silica, graphene oxide, and zinc oxide nanoparticles) have been successfully explored to develop functional antimicrobial films of chitosan. In a recent study, chlorogenic acid was loaded into halloysite nanotubes [48]. Chemically, chlorogenic acid is a naturally occurring bioactive agent, an ester of caffeic and quinic acids. The halloysite nanotubes were then incorporated within the chitosan/polycaprolactone blends. A chlorogenic acid-loaded halloysite nanotube of chitosan/polycaprolactone nanocomposite films was prepared through electrospinning. Though the inclusion of chlorogenic acid improved the thermal stability of the films (fibrous mats), the mechanical properties of the films were impaired. The increment in the thermal property was due to the increase in hydrogen bonding. The increased hydrogen bonding was related to the addition of chlorogenic acid. The synthesized films released the chlorogenic acid in a sustained manner. The presence of chlorogenic acid enhanced the antioxidant and antimicrobial properties of the nanocomposite films [48].

Metallic and metallic oxide nanoparticles have gained much attention in the last decade in regard to the development of active packaging systems. Zinc oxide nanoparticle (ZnONP) is one such type of nanoparticle. ZnONPs impart antimicrobial activity against a broad range of microbes. It can also improve the mechanical and barrier properties of biopolymeric films. The nanoparticle has been regarded as safe for human consumption by The United States Food and Drug Administration (FDA) [49]. In [49], authors have reported the synthesis of chitosan and ZnONP-based nanocomposites. The nanocomposite films were loaded with gallic acid. Chemically, gallic acid is 3, 4, 5-trihydroxy benzoic acid. It is a polyphenolic compound. Intrinsically, gallic acid is an antimicrobial and antioxidant agent. These properties of gallic acid were exploited to develop active functional packaging materials. In the study, the nanocomposite films of chitosan were prepared using the ZnONP-loaded gallic acid system [49]. The loading of the ZnONP with gallic acid allowed the combination of the beneficial properties of both ZnONP and gallic acid. The prepared nanocomposite films exhibited excellent antimicrobial and antioxidant properties over the pristine chitosan films. In [50], ZnONP and silver nanoparticle-based chitosan nanocomposites were prepared. The antimicrobial activity of the films gaged against *S. aureus* and *E. coli* by disc assay and cell-growth curve analysis. Both the films were found to be active against the named bacteria. The silver and chitosan nanocomposites showed better antimicrobial activity over the ZnONP-based nanocomposite. Similar to the ZnONP, magnesium oxide nanoparticles (MgONPs) have also been explored to develop nanocomposites for active food packaging materials [51]. In the study, the authors have synthesized nanocomposite films of MgONP and carboxymethyl chitosan. The incorporation of MgONP within the chitosan-derivative matrix enhanced the thermal stability, UV-barrier property, and water insolubility, making films suitable for water-rich food products. The films showed excellent antimicrobial activity against *L. monocytogenes* and *S. baltica* [51]. Recently, silver nanoparticle-embedded nanocomposites, wherein a chitosan/PVA blend was used as the polymer matrix, have been synthesized in [52]. The chitosan was used as the stabilizing agent of the silver nanoparticles. The nanocomposite matrices prepared through electrospinning showed antimicrobial activity against *L. monocytogenes* and *E. coli*. The films were also evaluated for their ability to increase the shelf life of meat products. The study results suggested that the composite films protracted the shelf life of the meat products by one week. In another study, the silver-loaded nano-titania was then used as the filler material for the synthesis of fish gelatin and chitosan materials [53]. The interactions within the nano-composite films were improved by the silver-loaded nano-titania. The nano-composite films were expected to perform well as active packaging materials.

**Figure 20:**
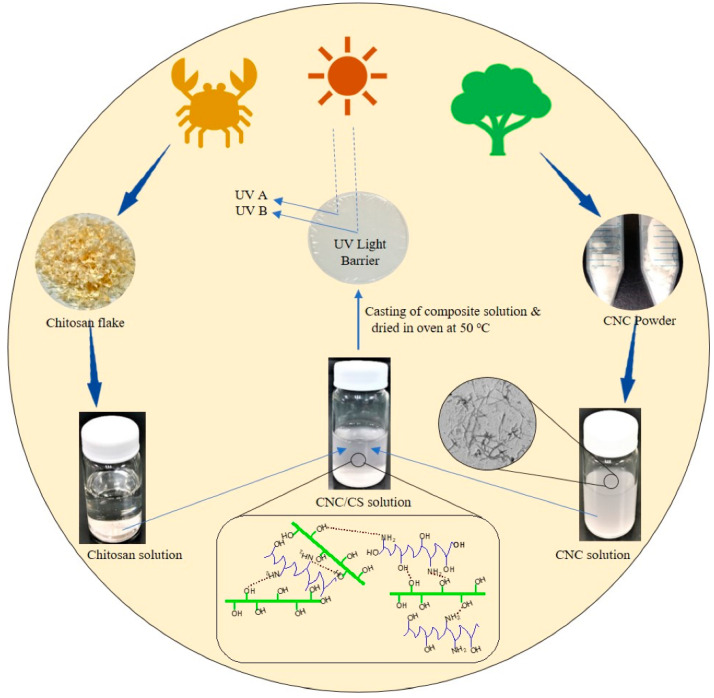
Overview of the methodology for the synthesis of CNC/chitosan nanocomposite films (taken from [56] under Creative Commons License).

The use of natural polymer-based nanoparticles allows for the improvement of the properties of the chitosan films without compromising the biodegradability of the films. This is because natural polymers are inherently biodegradable. Chitosan is readily compostable, and, therefore, their disposal is not harmful to nature [54]. The degradation of chitin and chitosan highlights their opportunity for recycling. One recently explored natural polymer-based nanoparticulate matter includes cellulose nanocrystals (CNCs) (Figure 20). The development of chitosan and CNCs have been proposed as active pads for meat packaging applications [55]. The inclusion of CNCs improved the pristine films’ mechanical properties, thermal stability, and oxygen barrier properties. Interestingly, the moisture retention ability of the films remained unhindered. The nanocomposites showed wide antimicrobial activity against Gram-positive and Gram-negative bacteria and fungi (*Candida albicans*). Most importantly, the packages were able to prolong the shelf life of the meat products under refrigeration by impeding the spoilage of the meats.

One of the developing techniques for fiber preparation for possible food, biomedical, and packaging systems is electrospinning. Arietta et al. [57] prepared biocomposite using chitosan and catechin, based on poly(lactic acid) (PLA) and poly(hydroxybutyrate) (PHB). The results showed that incorporating chitosan into the blend matrix affects the mechanical properties, i.e., tensile strength, elastic modulus, and elongation. Furthermore, the prepared biocomposite films were disintegrated in composting conditions. Therefore, these structures are applied to a new, sustainable, end-life packaging system. The electrospinning method can also be used for bioscaffold nanocomposites preparation. Toloue et al. [58] studied poly (3-hydroxybutyrate)-chitosan/alumina nanowires composition for possible bone tissue engineering applications. Here, the desirable properties of bioscaffolds were enhanced by the addition of alumina. Synergic effects were observed during the evaluation of mechanical properties and cell behavior studies, and bioactivity tests. Electrospinning can be successfully applied for porous materials preparation, as well as membranes. Studies presented in [59] showed that chitosan NPs into PLA enhanced the antibacterial performance of the obtained fibrous membranes. 

### 4.3. Chitosan as Nanofillers

Most of the biopolymers that are used for packaging materials are inherently weak in their physical, mechanical, and thermal aspects. Blending these polymers with nanofillers is one of the widely accepted approaches for overcoming the drawbacks mentioned above. The field of nanotechnology explores the utilization of materials having a nanometer size range for a large number of novel applications. Chitosan nanoparticles can be obtained from polymeric chitosan through approaches such as chemical crosslinking, precipitation, and microemulsion technique, etc. [60]. The integration of these nanoparticles in the biopolymeric matrix has improved the barrier properties for gas and water, elevated mechanical strength, and produced superior antimicrobial activity. Starch is a biodegradable thermoplastic polymer that is often used in the development of packaging material. The inclusion of chitosan nanofillers into the starch matrix improved the mechanical strength [61]. The possible reason behind this can be chitosan, which acted as a junction point in the starch matrix. The prepared film displayed sustainable behavior at higher temperatures, suggesting their potential role in heat-sealing packaging applications. Chitosan nanofibers are also known for their homogenous distribution in the matrices of many biopolymers. A gelatin-based film containing chitosan nanofiber and ZnO nanoparticles was synthesized for the safe packing of chicken fillet, considering the advantages of chitosan [62]. The organoleptic features of the packed chicken fillet were suitable for consumption [62]. In general, an increased amount of chitosan nanofillers results in increased intermolecular forces. Such an increase in intermolecular forces assists in decreasing the free volume of the film matrices [63], thereby forming a denser network structure with very low permeability. Chitosan nanofillers display a different attribute to the polymeric system compared to a bulk system made from chitosan. These additional attributes can be correlated to the promoted surface-to-volume ratio provided by the nanofillers. 

## 5. Conclusions

Over the last couple of decades, consumers have become highly concerned about consuming healthy foods and the storage of food products for longer durations. Food packaging systems play an essential role in keeping food safe for consumption for prolonged periods. Table 1 provides brief details on the uses of chitosan (source)/derivatives for the specific antimicrobial activity or food storage effectiveness. During the initial days, the industries preferred petroleum-based food packaging systems, which are non-biodegradable, to develop packaging materials due to their numerous beneficial properties and ease of production and handling of the final products. Unfortunately, petroleum-based food packaging systems are non-biodegradable, which makes them difficult to dispose of. Due to this reason, petroleum-based food packaging systems are creating environmental hazards. Hence, there has been a massive surge in research activities to develop biodegradable food packaging systems using biopolymers in the last decade. Polysaccharides and carbohydrate-based polymers, explored extensively due to their easy availability, making them economical raw materials. Among the different polysaccharides, the quest for developing chitosan-based packaging systems has expanded in multitude. This is because chitosan is obtained from the by-products of the seafood industry.

Further, chitosan has several inherent beneficial properties (e.g., antimicrobial, antioxidant, chelation, and film-forming properties) that provide active protection to food products. The antimicrobial and antioxidant properties can be enhanced by adding several additives (e.g., essential oils and natural extracts). The properties of the chitosan films can be improved either by blending with other polymers, such as gelatin and PVA, or by reinforcing the chitosan films (pristine of blends) with nanoparticulate systems (e.g., metallic, metallic oxide, or polymeric nanocrystals). It is important to note that chitosan and its derivatives have also found immense applications in cosmetics, pharmaceuticals, biomedical, and nutraceutical industries, apart from food packaging applications. The particular interest of researchers on chitosan, due to its specific antibacterial and antifungal characteristics, is also observed in dentistry, where innovative therapeutics are being sought.

Even though chitosan has several beneficial properties and has been used extensively in many industries, its solubility at physiological pHs is the major problem of chitosan. This can be overcome either by converting it into salt or water-soluble derivatives. Recently, low molecular-weight chitosans, which are soluble in water, have also been proposed. In this review, we have discussed the different methodologies for the synthesis of chitosan-based films. The coating methods employed to coat the fresh food products with chitosan and its blends and derivatives have also been discussed briefly. The chitosan-based films and coatings have been reported to significantly enhance the shelf life of the food products without compromising their quality. It is expected that chitosan and its derivatives will be commercially exploited to develop advanced food packaging systems soon.

## Figures and Tables

**Table 1:** Uses of chitosan (source)/derivatives for the specific antimicrobial activity or food storage effectiveness.

Matrix Material(s)	Source of Chitosan	Antimicrobial Activity	Food Storage Effectiveness	References
Chitosan	Larvae of housefly	*E. coli*, *Staphylococcus aureus*, *Salmonella typhimurium*, *Listeria monocytogenes*, and *Bacillus cereus*.	Including garlic oil into chitosan film acts as a physical and antimicrobial barrier to food contamination.	[64]
Chitosan	Larvae of housefly	*Rhizoctonia solani*, *Thanatephorus cucumeris*, *Sclerotina sclerotiorum*, *Curvularia lunata*	Natural antioxidant for functional food products and holds the antifungal property.	[65]
PVA, Chitosan, and silver nanoparticle	-	*E. coli* and *L. monocytogenes*	PVA-CH-Ag composite nano-layer displayed better organoleptic qualities, such as visual appearance and smell of packaged meat.	[52]
Chitosan-TiO2	Shrimp shells	*E. coli*, *S. aureus*, *C. albicans*, and *A. niger*	Chitosan-TiO2 film successfully shields red grapes from microbial spoilage and extends their shelf life.	[66]
Chitosan, apple peel polyphenols	-	*E. coli*, *B. cereus*, *S. aureus* and *S. typhimurium*	The incorporation of polyphenols in the chitosan film can show an enhanced shelf life of food products	[67]
Binary grafted chitosan film with acrylamide and acrylonitrile	-	*Staphylococcus aureus*, *Escherichia coli*, *Pseudomonas aeroginosa*	Protect apple and guava from microbial spoilage	[68]
Cellulose/titania/chitosan	Shrimp shells	*E. coli* and *S. aureus*	The formed hybrid material has enhanced antibacterial property essential for food packaging	[69]
Cellulose, chitosan		*E. coli* and *Staphylococcus aureus.*	Better performance than traditional polyethylene packaging of sausages	[70]
Carboxymethyl cellulose, 2-N-Hydroxypropyl-3-trimethylammonium chloride chitosan	-	*E. coli* and *Staphylococcus aureus.*	The blended films have shown extended shelf life for packaging of banana	[71]
PEGylated chitosan	-	*E.coli*	Mechanical and thermally stable films for food packaging enhances anti-bacterial property	[72]

## Data Availability

All collected data were presented in the manuscript.
